# The tree-ring width and interval trend values as indicators of sensitivity to temperature and precipitation in different provenances of European larch

**DOI:** 10.1038/s41598-025-85652-5

**Published:** 2025-01-11

**Authors:** Norbert Szymański, Sławomir Wilczyński, Jan Kowalczyk, Wojciech Kowalkowski

**Affiliations:** 1 Department of Forest Ecology and Silviculture, University of Agriculture in Krakow, al. 29 Listopada 46, Krakow, 31-425 Poland; 2 Department of Forest Ecosystem Protection, University of Agriculture in Krakow, al. 29 Listopada 46, Krakow, 31-425 Poland; 3 Department of Silviculture and Genetics of Forest Trees, Forest Research Institute, Sękocin Stary, ul. Braci Leśnej 3, Raszyn, 05-090 Poland; 4 Department of Silviculture, Poznań University of Life Sciences, ul. Wojska Polskiego 71A, Poznań, 60-625 Poland

**Keywords:** Larix, Radial growth, Pointer year, Resilience, Drought, Poland, Forestry, Plant stress responses, Forest ecology, Forestry

## Abstract

The study assessed the sensitivity of 20 provenances of European larch (*Larix decidua* Mill.) growing at provenance experimental trials located in lowland (Siemianice) and upland (Bliżyn) climate in Central Poland to air temperature and precipitation, including drought. The measure of the tree’ sensitivity was their radial growth reactions, i.e. changes in the radial growth in years 1971–2015. We found that rainwater supplies in a soil stored in autumn of the previous year, length of the growing season and thermal conditions in its beginning, as well as thermal and moisture conditions of the year of tree ring formation had a significant impact on the wood volume formed by the larches, regardless of their origin and climatic region in which they grew. The degree of homogeneity of tree’ radial growth reactions to precipitation deficit and high temperature was the lowest in a warmer and drier climate in the lowlands in Central Poland. Larch provenances with the lowest and the highest values of drought resilience components (resistance, recovery, resilience, relative resilience of radial growth) originated in different regions of Poland. Greater resistance to drought was observed in larch provenances growing at the trial located in the uplands. The relative resilience index seems to be the most helpful in predicting the future radial growth reactions of the studied provenances, and consequently their viability and survival, as this index showed the highest variability among trees of a given provenance and was most often significantly different between pairs of provenances.

## Introduction

Due to a constant increase in air temperature and changes in precipitation distribution, climate models predict an increase in the frequency of extreme events, i.e. droughts, extreme temperatures and intense storm precipitation^1–3^. For example, in recent decades, the length and intensity of summer droughts in Central Europe have doubled^4^, making them one of the main challenges for forest management^5,6^. Long-term precipitation deficit limits the growth of trees^7,8^ and often contributes to their dying^9–11^. In order to ensure productive and viable tree stands, their resistance to various harmful environmental factors should be increased, i.e. through the selection of appropriate tree species^12^ improvement cutting^13^ and genetic selection^14^. For example, the radial growth response of trees to drought depends on a tree species^15,16^, biosocial position of tree in a stand^12,17^, age^18,19^, habitat conditions^8^ and tree origin^20–29^.

In the face of many threats, provenance experiments are helpful, as they allow us to indicate which tree populations are more resistant to adverse meteorological conditions, mainly droughts. However, trees of a given population may differ in the radial growth reactions, resulting from individual genetic predispositions. Therefore, the chronologies of interval trend indices are often used to assess the mass and common growth reactions of trees. The interval trend values can be straightforwardly interpreted in contrast to the abstract values of other popular types of tree-ring index chronologies^30^. This indicator shows the percentage of trees with a specific growth response, i.e. an increase or reduction in growth, to the pressure of a specific climatic element. Elements determining the occurrence of larch pointer years were successfully identified by analysing climatic conditions prevailing in pointer years and in the years immediately preceding them^31–34^.

Larch is one of the most high-transpiration species and uses water inefficiently^35–39^. Therefore, long periods of precipitation deficit can be a highly destructive factor for larch, which disturbs a water balance in a tree, weakens its growth and even causes death. Previous studies on the intraspecific variability of European larch indicate its sensitivity to precipitation deficits, especially during the growing season^34,40–43^. In addition, European larch is characterized by high spatial and temporal variability in its climate-growth relationships^33,44–50^.

The impact of droughts on the growth condition (vitality) of trees can be assessed, among others, using the resilience components proposed by Lloret et al.^18^. One of them is the estimation of the resistance index (Rt), which consists in the lack of tree’s reaction to a stress factor (e.g. drought). On the other hand, recovery (Rc) is the process of rebuilding the radial growth after the period of stress, while resilience (Rs) is the ability of tree to recover the ring growth from before a disturbance. These indices were used to investigate the drought resilience, e.g. in yellow pine^18^, Norway spruce^51^, Scots pine^12^, Douglas fir and silver fir^15^, Australian cedar and Indian mahogany^16^, Scots beech^52^, *Picea crassifolia*^53^, English oak and Sessile oak^54^, Siberian larch^55^, Austrocedrus chilensis^56^, Chinese pine^57^, and black pine^58^.

The aim of the study was to assess the sensitivity of European larch trees of 20 provenances to thermal and moisture conditions, and droughts, on provenance experimental trials located in two Polish regions differing in climatic conditions – in lowlands and uplands.

On the basis of the current knowledge about the sensitivity of European larch to the climatic factor, we have adopted the following hypotheses:

### H1

The primary factor influencing the radial growth of larch, irrespective of tree origin or experimental plot location, is the length of the growing season shaped by an air temperature in its beginning, and thermal and moisture conditions during the growing season;

### H2

On both experimental plots, larches with high drought resilience components originate from lowland regions with low precipitation, while larches with low drought resilience components are from upland regions with abundant precipitation;

### H3

Larch provenances growing in colder, precipitation-rich upland plots, which are not frequently exposed to drought, exhibit lower drought resilience components than those growing in lowland plots that are warmer and have less precipitation.

## Methods

**Fig. 1:**
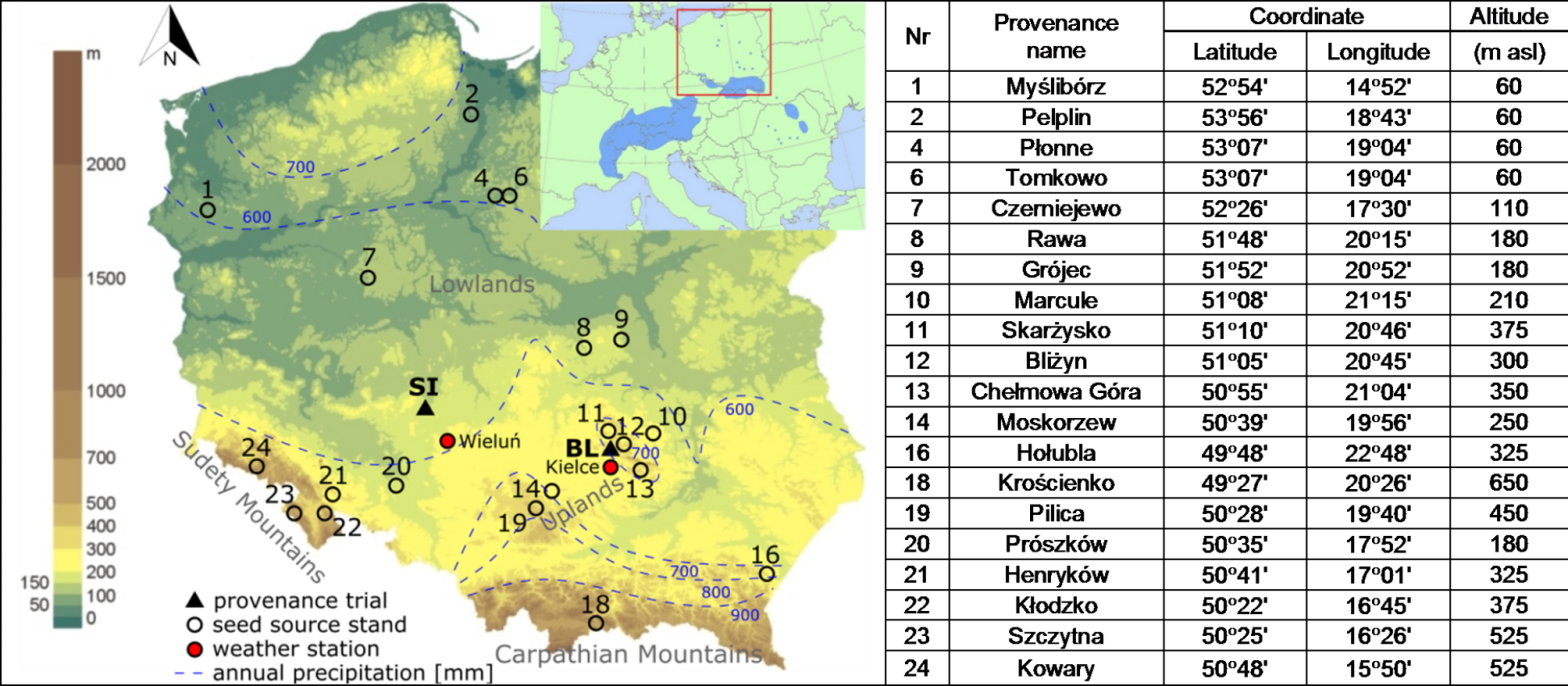
Locations of the seed source stands, the provenance trials in Siemianice (SI), Bliżyn (BL) and the weather stations. Distribution map of European larch (at the top right)^59^.

The study was carried out at two experimental trials where 20 Polish larch provenances were grown (Fig. 1). These trials were located in the lowlands in 1967 (Siemianice – SI; 51°13’N, 18°03’E, 170 m asl) outside the natural distribution range of European larch and in the uplands in 1968 (Bliżyn – BL; 51°02’N, 20°40’E, 305 m asl) within this range (Fig. 1).

For each provenance, a total of 20 larch trees aged 51–53 years were drilled twice from E and W stem direction at a height of 1.3 m above the ground using increment borer during the period 2015–2017. The sanded cores were scanned using an optical scanner (2400 DPI). Tree-ring widths were measured and cross-dated using CooRecorder and CDendro 7.8 computer software^60^. The cross-dating were examined using the COFECHA program^61^. Two series from a given tree were averaged. Consequently, each larch tree was represented by an individually tree-ring width chronology for the period 1970–2015. One chronology of interval trend indices (IT) for each larch provenance at each provenance trial was developed on the basis of 20 tree chronologies (1)^30^.

1 $$\:{IT}_{i}={m}_{i}\bullet\:{{k}_{i}}^{-1}$$

where *m* is the number of trees that in a given year *i* showed an increase in the ring-width compared to the previous year plus half of the trees that had the same ring-width as in the previous year; k – is the number of all analyzed tree chronologies.

One IT chronology for each provenance trial was developed on the basis of 400 tree chronologies. The time period common to all the IT chronologies used in the study was 1971–2015. The above-mentioned IT chronologies describe the degree of similarity of radial growth responses (an increase or decrease in growth) in larch of a given provenance and in all trees at a given provenance trial.

In order to determine differences between the IT chronologies of individual provenances and between the study trials, a grouping method was carried out using Ward’s hierarchical cluster analysis (distance: $\:1.0-r$ (the Pearson correlation coefficient)). Then, pointer years were determined for both experimental trials on the basis of IT values from 400 individual chronologies. It was assumed that a positive pointer year is the year in which IT value was higher than 0.9, i.e. at least 90% of trees increased a ring-width compared to the previous year. In a negative pointer year, IT value was lower than 0.1, i.e. at least 90% of trees reduced a ring-width compared to the previous year^62^. Climatic conditions in positive and negative years were then compared on basis of differences between monthly precipitation and temperatures^34^. In addition, a cluster analysis was performed on the series of correlation coefficients, which were calculated between the IT chronologies and mean monthly air temperatures and monthly precipitation totals.

We assessed the radial growth response of each larch provenance to drought using the indices of resistance (Rt), recovery (Rc), resilience (Rs) and relative resilience (RRs)^18,51^. The Rt is the measure of reduction in the growth caused by a drought. It is calculated as the proportion of mean ring-width in the year of drought (Dr) and the mean ring-width of the 3 years before the drought (PreDr): Rt = Dr / PreDr. Rt = 1 means that tree is fully resistant to drought. The lower the Rt value, the lower the drought resistance of tree. The Rc is the measure of tree’s ability to recover radial growth capacity after a drought. This is the proportion of mean increment of the 3 years after a drought (PostDr) and increment of the drought year (Dr): Rc = PostDr / Dr. Rc = 1 indicates the maintain of low level of radial growth after drought, and Rc < 1 indicates a deepening reduction in growth after a drought, while Rc > 1 indicates that a tree regained growth capacity higher to the level which reached in the year with a drought. The Rs describes the ability of tree to regain a pre-drought growth level. This is the proportion of the mean increment of the 3 years after a drought (PostDr) and the mean increment of the 3 years before the drought (PreDr): Rs = PostDr / PreDr. Rs > 1 indicates that a tree fully recovered growth capacity after a drought and is highly flexible in response of adverse growing conditions. Rs < 1 indicates the low adaptability of tree to changing growing conditions and indicates a long-term reduction in growth after a drought. The RRs determine how quickly tree is able to regain the level of pre-drought radial growth, taking into account a reduction in growth in the year of drought. It is calculated according to a formula: RRs = ((PostDr – Dr) / (PreDr – Dr)) (1 – (Dr/PreDr)). The RRs index are interpreted in the same way as in the case of the Rs index, but it may have negative values if a mean increment of the 3 years after a drought is lower than a increment in the drought year. A high value of the resistance index (Rt) decreases the relative resilience (RRs), while a low one increases it.

The assessment of hydrothermal conditions and the identification of drought years in the period 1970–2015 were carried out on the basis of Sieljaninov’s hydrothermical index (2), which is expressed as the proportion of 10 times the values of monthly precipitation and the monthly total of mean daily air temperatures for the period in which a mean daily temperature exceeded 10 °C^63^. The lower the HTC value, the drier the conditions.

2 $$\:HTC=\frac{10{\sum\:}_{i=1}^{n}{P}_{i}}{{\sum\:}_{i=1}^{n}{t}_{i}}$$

where: n – length of the analyzed period (days),

P_i_ – total precipitation on the day *i* (mm),

t_i_ – mean daily air temperature on the day *i* (°C),

HTC < 0.4 extremely dry year, 0.4 < HTC ≤ 0.7 very dry, 0.7 < HTC ≤ 1.0 dry, 1.0 < HTC ≤ 1.3 fairly dry, 1.3 < HTC ≤ 1.6 optimal, 1.6 < HTC ≤ 2.0 moderately humid, 2.0 < HTC ≤ 2.5 humid, 2.5 < HTC ≤ 3.0 very humid, HTC > 3.0 extremely humid).

**Fig. 2:**
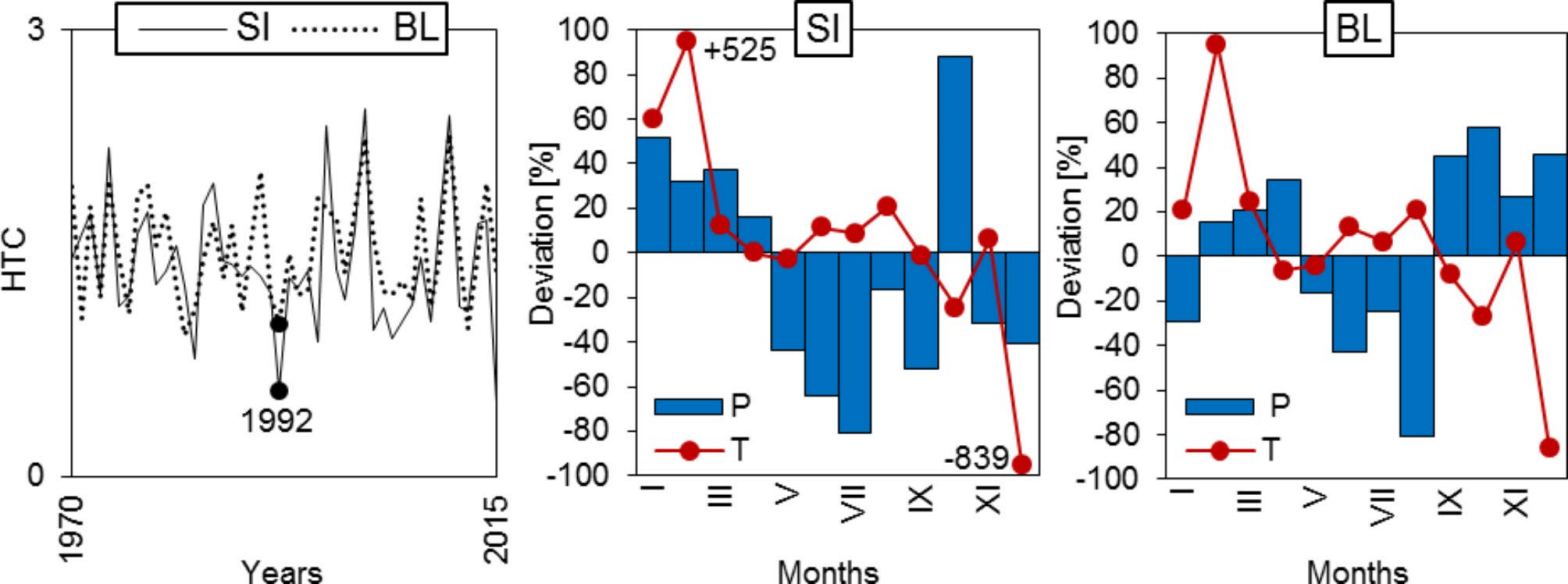
Sieljaninov’s hydrothermical index (HTC), deviations (%) of monthly mean air temperatures (T) and precipitation totals (P) in 1992 from the climate norm (1970–2015) in the trials in Siemianice (SI) and Bliżyn (BL).

The year of the lowest sum of HTC value from two experimental trials was analysed (SI = 0.6, BL = 1.0) (Fig. 2), assuming that in this year occurred at least 25% reduction in the radial growth compared to the mean increment of the 3 previous years. In this way, the certainty that the decline in the growth was related to droughts was increased^18^. It turned out that on both trials it was 1992. The diagrams of deviations of thermal and moisture conditions from the climatic norm confirmed that this year was a particularly warm and dry in the growing season (Fig. 2). This year was the worst in terms of precipitation and temperature in both regions. In the SI trial, the very dry years were 1983, 2015, and only fairly dry years occurred in the BL: 1971, 1976, 1982, 1983, 1988, 2008, 2012.

In order to determine whether larch provenances differ in sensitivity to a drought, the ANOVA and then post-hoc Tukey’s HSD test (α = 0.05) for the Rt, Rc, Rs and RRs indices was used. HSD test categorized the index value into homogeneous groups. From these groups, were selected and showed the provenances whose index value was significantly different from the provenance with the lowest index values, and the provenances whose index value was significantly different from the provenance with the highest index values^64^.

The values of climatic parameters used in the analyses came from the nearest IMGW-PIB meteorological stations with full data set: Wieluń (51°12’40"N, 18°33’28"E; 201 m asl) and Kielce (50°48’37"N, 20°41’32"E; 261 m asl)^65^.

### Climate

**Fig. 3:**
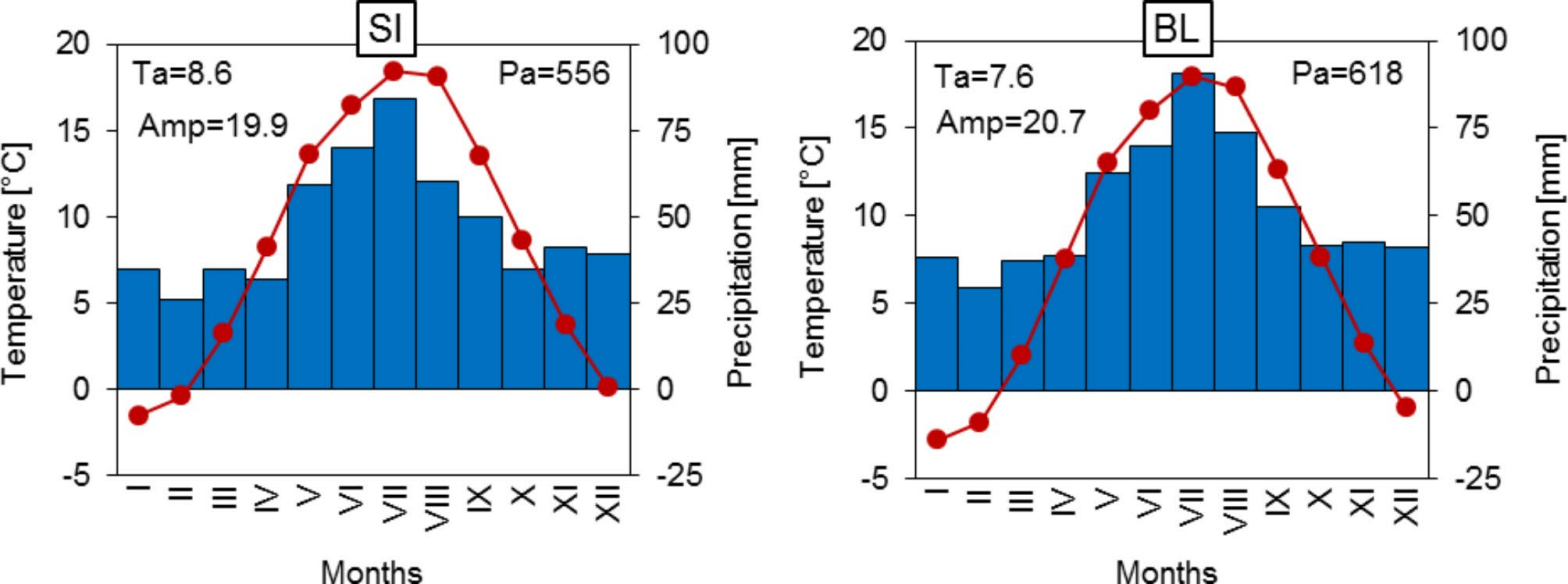
The climate diagrams for the trials in Siemianice (SI) and Bliżyn (BL) for the period 1970–2015. Ta – mean annual air temperature, Amp – mean annual air temperature amplitude, Pa – mean annual precipitation total.

The two experimental trials differed in climatic conditions. The SI trial is located in a lowland area with a warmer and drier climate compared to BL. The mean annual temperature (Ta) was 8.6 °C and the mean annual precipitation total (Pa) was 556 mm for the period 1970–2015 (Fig. 3). The BL trial was situated in an upland with a colder and more abundant in precipitation climate (Ta = 7.6 °C, Pa = 618 mm) than in SI. The annual air temperature amplitude (Amp = 20.7 °C) in BL trials indicates that there was more continental climate than in SI. In SI trial, located further west, the annual amplitude was 19.9 °C. On average, the highest precipitation in SI and BL occurred in July (84 and 91 mm, respectively). The lowest precipitation totals on both trials occurred in February (SI = 26 mm, BL = 29 mm).

## Results

### Radial growth reactions of larches at provenance trials

**Fig. 4:**
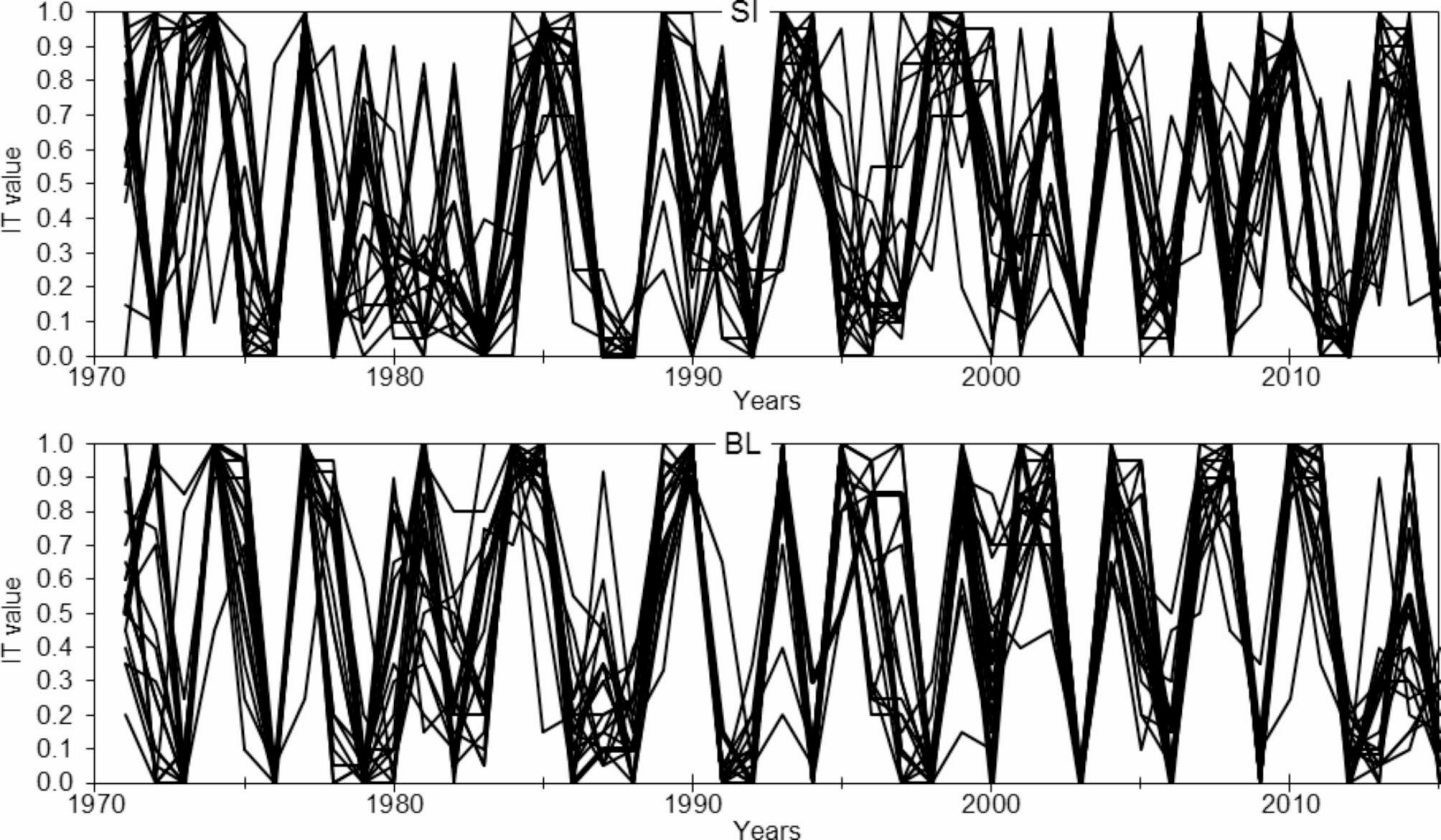
IT chronologies of larch provenances growing at the trials in Siemianice (SI) and Bliżyn (BL).

**Fig. 5:**
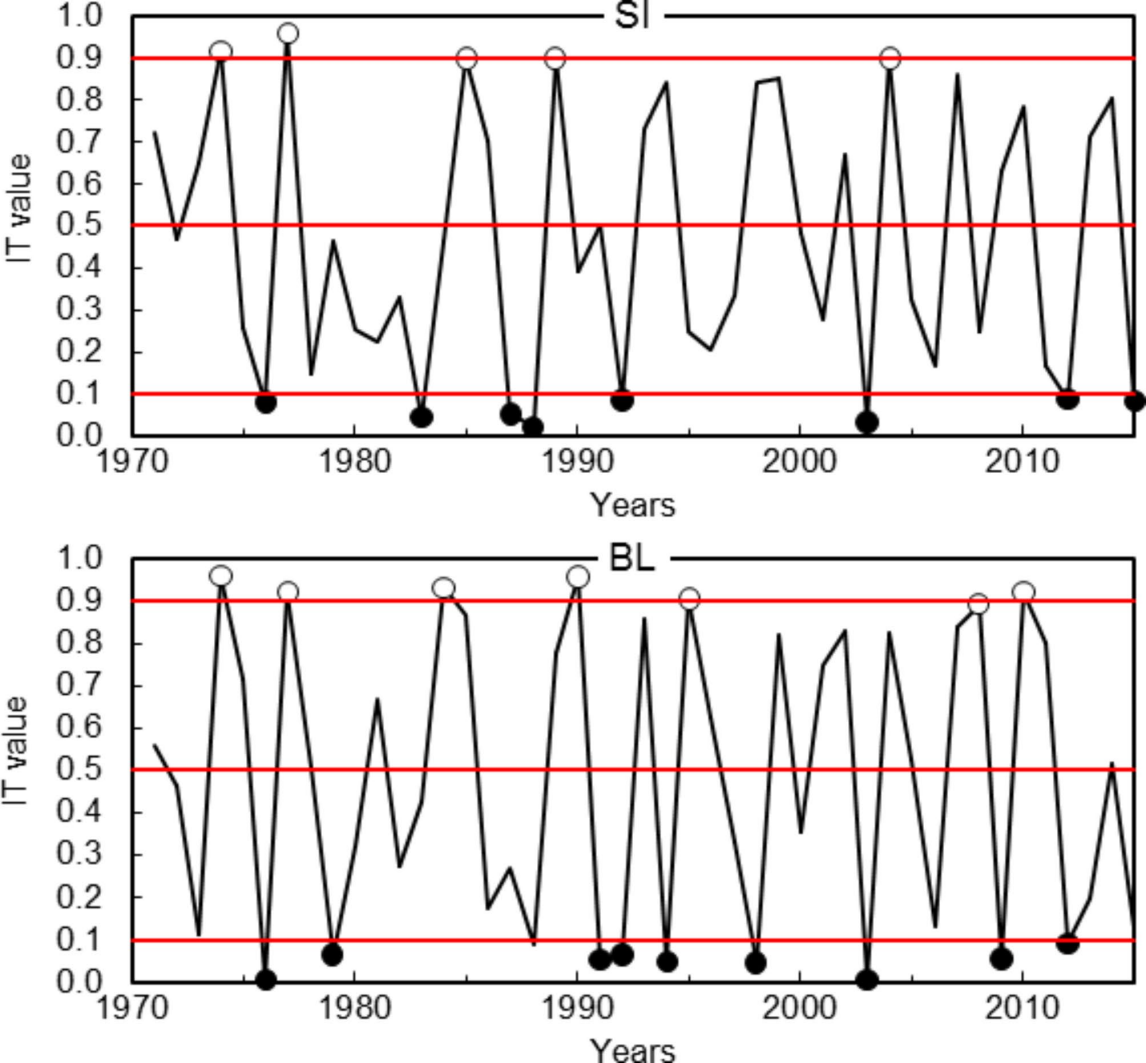
IT chronologies for larches from the trials in Siemianice (SI) and Bliżyn (BL), and positive (white dots) and negative (black dots) pointer years.

The IT chronology courses for 20 larch provenances are strongly differentiated in both trials (Fig. 4). There are no years in which all trees growing at a given trial have the same radial growth reaction (IT = 0 or 1). This reaction is understood here as the proportion value of the number of trees with the specific direction of the year-to-year change in the size of increment. Positive reactions at SI trial are the most in 1977 (IT = 0.960), and negative reactions are the most in 1988 (IT = 0.021). At BL, respectively 1990 (IT = 0.957) and 1976, 2003 (IT = 0.005) (Figs. 4 and 5).

**Fig. 6:**
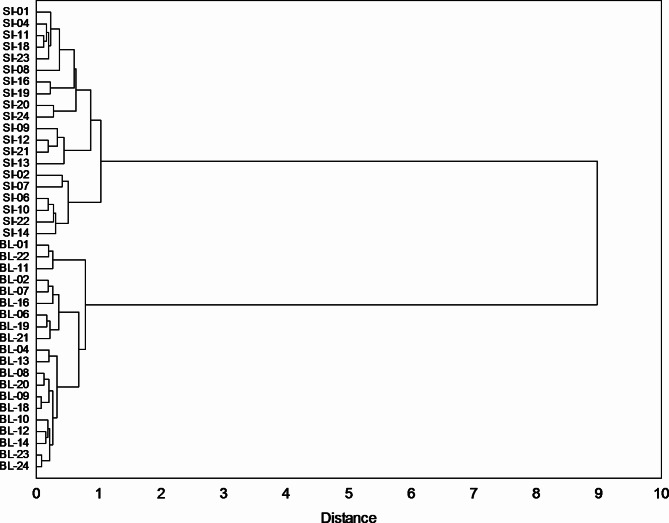
Grouping of IT chronologies of larch provenances growing at the trials in Siemianice (SI) and Bliżyn (BL) in terms of radial growth reactions of trees.

The cluster analysis (CA) of IT chronology identified two main groups of larch provenances: The first consisted of chronologies of provenances growing at the SI trial, whereas the second one the chronologies of provenance growing at BL trial (Fig. 6). Thus, the radial growth reactions of larches growing at both experimental trials were different and independent of the region of larch origin. Therefore, it can be tentatively assumed that a factor differentiating the chronologies between the trials was specific climatic conditions prevailing in these trials. In addition, we found for both experimental trials that it was not possible to distinguish the subgroups of larch provenances associated with a specific region of their origin (Fig. 6).

**Fig. 7:**
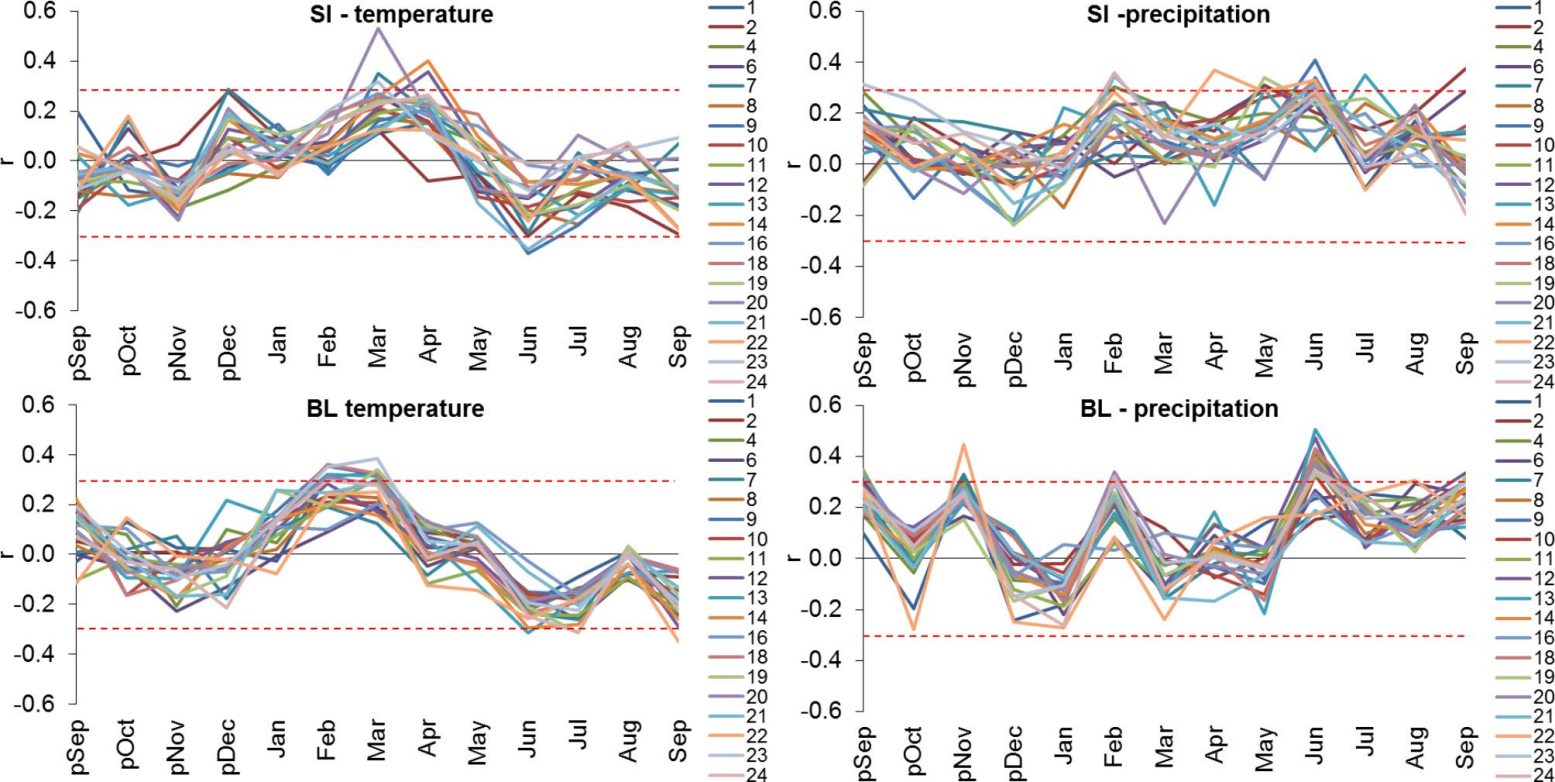
Series of correlation coefficients (c) for 20 larch provenances at the trials in Siemianice (SI) and Bliżyn (BL), as calculated between the IT chronologies and mean monthly air temperature and total monthly precipitation for 13 consecutive months from September of the previous year (pSep) to September of the year of tree ring formation (Sep) for the period of 1971–2015. Critic values for α = 0.05 – dashed lines.

In order to confirm the assumptions that the factor differentiating the interannual radial growth variation of European larch at both trials is the climatic conditions prevailing there, the Pearson correlation coefficients between IT chronologies and the monthly values of temperature and precipitation were first calculated (Fig. 7).

The course of correlation coefficients series for 20 provenances in the case of temperature was similar. A slightly greater variation in the course of series was observed in the case of precipitation, especially at the BL trial (Fig. 7).

**Fig. 8:**
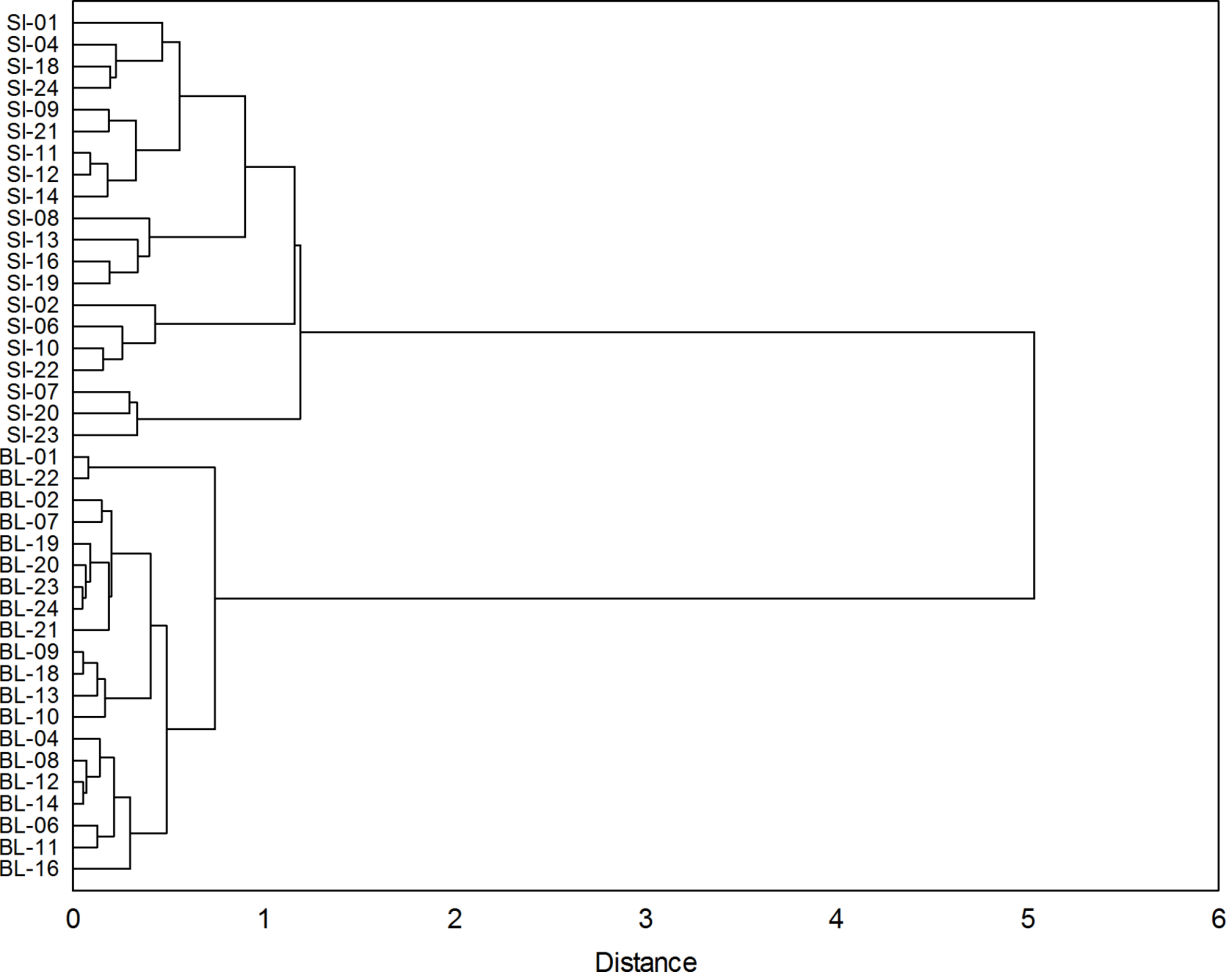
Grouping of the series of correlation coefficients of the larch provenances (SI - Siemianice, BL - Bliżyn) (see Fig. 7). Cluster analysis was used – Ward’s method and 1-r Pearson’s distance measure.

Then, a cluster analysis was performed in which variables were the above-mentioned series of correlation coefficients. This analysis grouped larch provenances into two clusters associated with the experimental trial (Fig. 8). The results are very similar to the results of the cluster analysis for the IT chronology (see Fig. 6).

Subsequently, on the basis of the IT chronology course developed from all tree-ring width chronologies (*n* = 400), 5 positive and 8 negative pointer years were found at the SI trial (Fig. 5). We were found more pointer years at the BL trial than at SI, respectively 7 and 10.

**Fig. 9:**
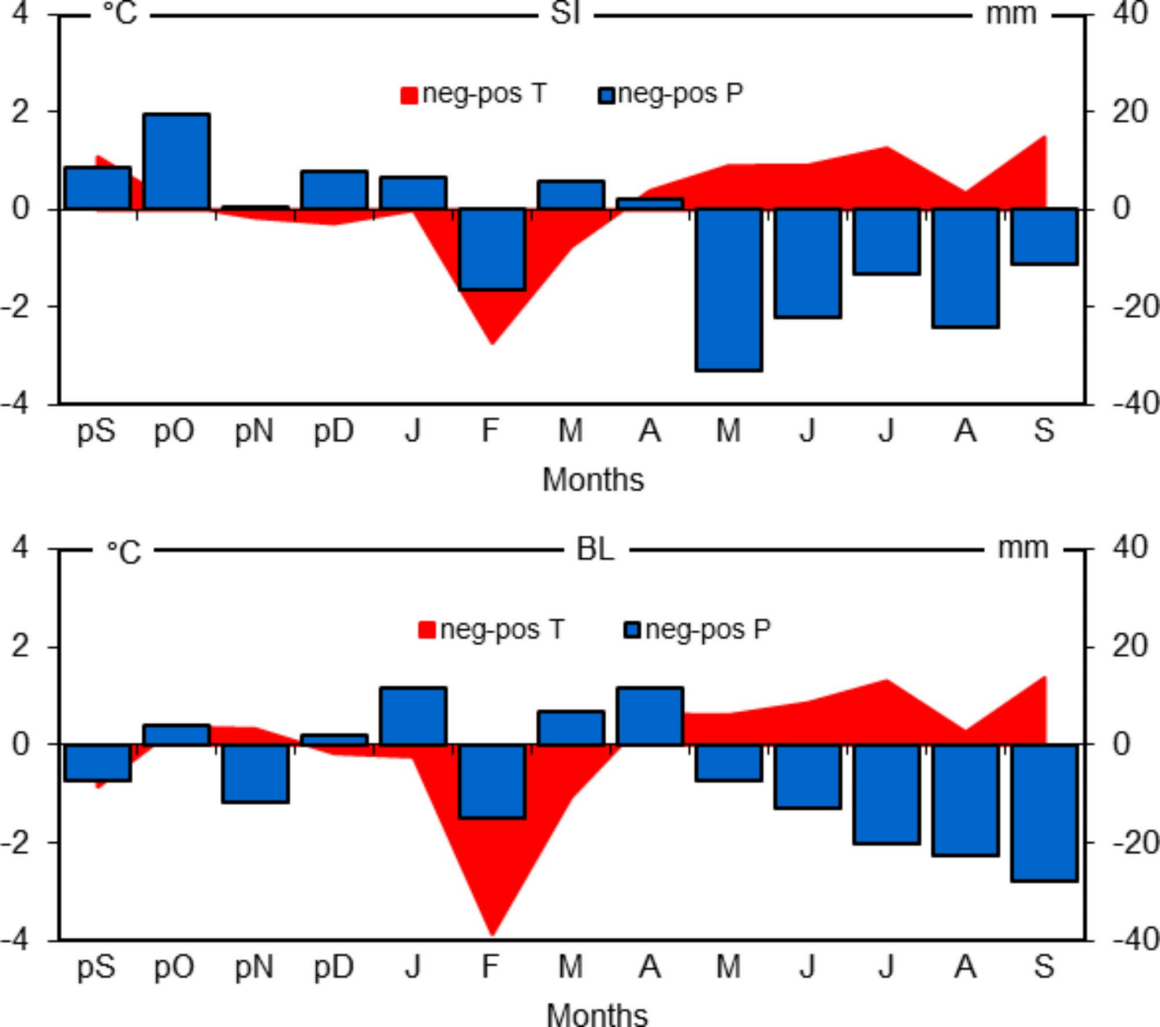
Differences between mean monthly temperature (T) and precipitation (P) in negative (neg) and positive (pos) pointer years of larches from the trials in Siemianice (SI) and Bliżyn (BL) (values in neg. were subtracted from pos.)

In contrast to the BL trial, February in the negative pointer years was cold and dry at SI. The growing season (May-September) was warm and dry at the SI trial in contrast to BL (Fig. 9). Similar conditions occurred in larch pointer years at BL.

### Differences in tree resilience to drought between trials and provenances

**Fig. 10:**
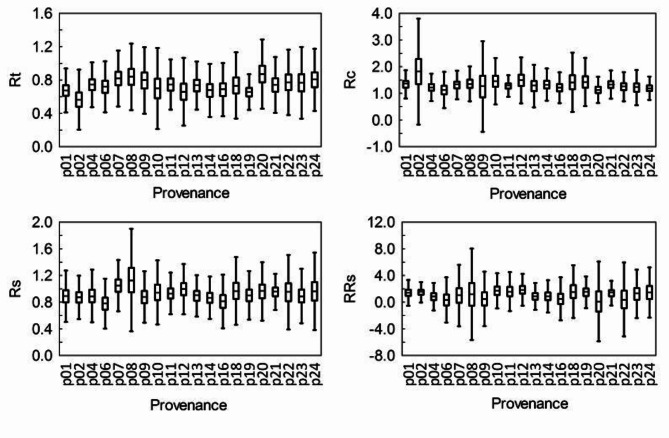
Resistance (Rt), recovery (Rc), resilience (Rs) and relative resilience (RRs) index of the radial growth of larch provenances after the 1992 drought in Siemianice. The bars – 95% confidence level. The vertical lines – one standard deviation from the mean.

**Fig. 11:**
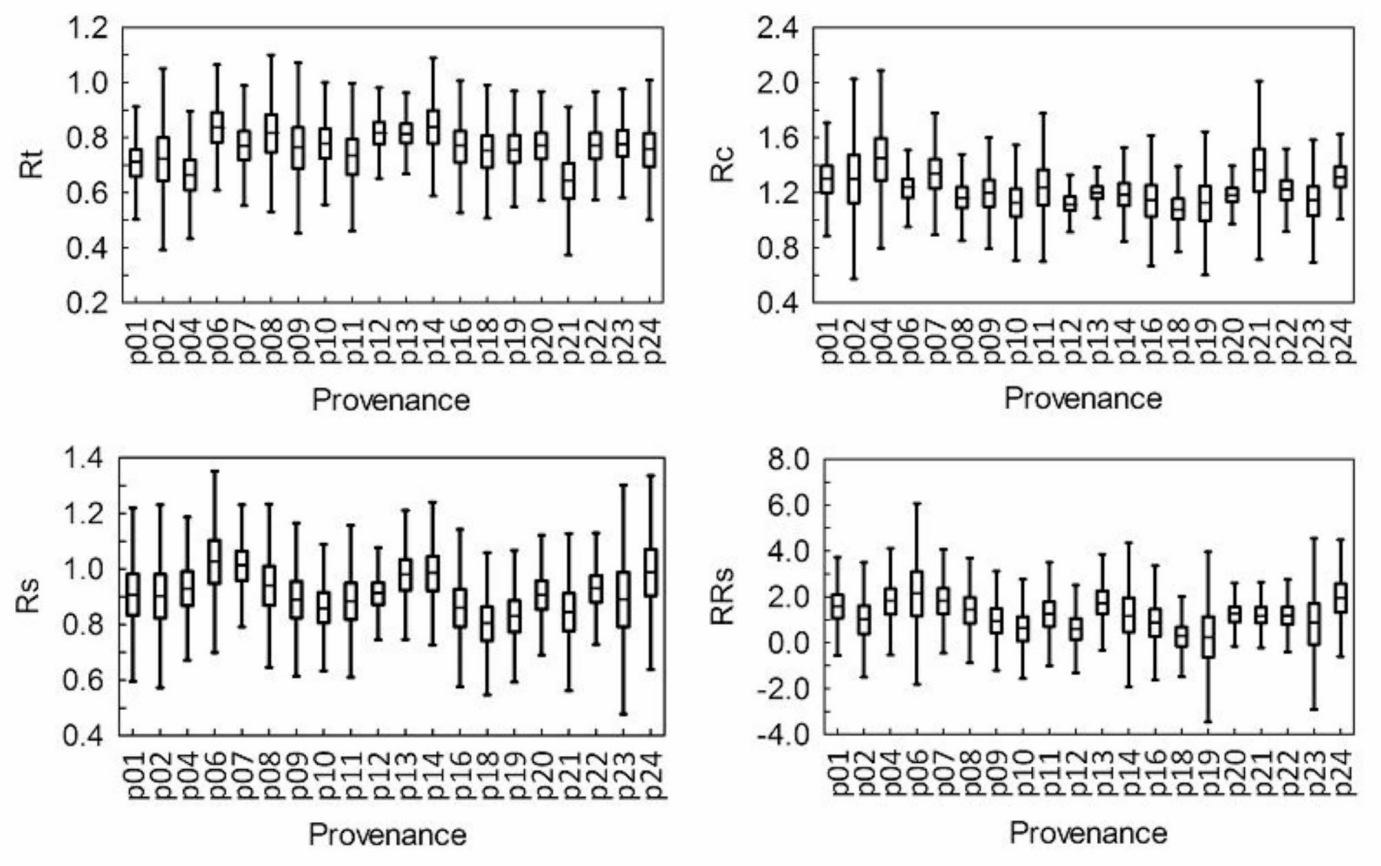
Resistance (Rt), recovery (Rc), resilience (Rs) and relative resilience (RRs) index of the radial growth of larch provenances after the 1992 drought in Bliżyn. The bars – 95% confidence level. The vertical lines – two standard deviation range from the mean.

At both SI and BL, the lowest values of resistance (Rt), recovery (Rc), resilience (Rs) and relative resilience (RRs) were observed across different provenances (Figs. 10 and 11). Similarly, the highest values of these indices varied by provenance.

At both trials, we also found statistically significant differences in the values of the above-mentioned indices between the provenances (Table 1). At SI, the least drought resistant larches came from Pelplin (p2), i.e. a northernmost seed source stand in Poland – the area with relatively high precipitation. The most drought resistant larches came from Prószków (p20) in the South-western Polish lowlands, i.e. from the area with relatively low precipitation (Table 1). At SI, the provenance with the worst recovery index after drought was no. 20, and the best was provenance no. 2. The lowest resilience indices (Rs, RRs) was observed in the northern Polish provenances “Płonne” (p6) and “Prószków” (p20), while the highest resilience indices was observed in the central Polish provenances “Rawa” (p8) and “Bliżyn” (p12). Taking into account all growth indices, the central Polish provenances are the best to survive the drought period at SI, and the least of the northern Polish provenances.

At BL, the least drought resistant (Rt) was the provenance “Henryków” from the Sudety Mountains (p21), and the most was upland provenance “Moskorzew” (p14), located in this climatic region of Poland, as the experimental trial (Table 1). The worst in terms of the recovery (Rc) and resilience (Rs, RRs) indices of growth was the mountain provenance “Krościenko” (p18) from the highest altitude of seed source stand, and the best was the northern Polish provenance “Tomkowo” (p6). At BL, provenances from the Northern Poland and central uplands are the best at coping with drought periods, in contrast to mountain provenances.

**Table 1:** The provenances (p) of larch with the lowest (min) and highest (max) values of resistance (Rt), recovery (Rc), resilience (Rs) and relative resilience (RRs) indices in 1992 in Siemianice (SI) and Bliżyn (BL).

Index	SI	BL
Rt_min_	p2 (4–9, 20–24)	p21 (2, 6–8, 12–14, 22, 23)
Rt_max_	p20 (1, 2, 19)	p14 (1,4, 21)
Rc_min_	p20 (1, 2,12)	p18 (1, 4–7, 13, 21, 24)
Rc_max_	p2 (4, 6, 11, 16, 24)	p6 (1–8, 12, 21, 22)
Rs_min_	p6 (7, 8, 12, 18, 19, 21)	p18 (4–8, 12–14, 22, 24)
Rs_max_	p8 (2, 6, 14, 16)	p6 (9–12, 16–19, 21)
RRs_min_	p20	p18 (1, 4–8, 11, 13, 20–22, 24)
RRs_max_	p12 (4, 6, 13)	p6 (1, 12, 18, 19)

The numbers of provenances whose index values are significantly (*p* < 0.05) higher than the provenance with the lowest index value and significantly (*p* < 0.05) lower than the provenance with the highest index value are given in brackets. Tukey’s HSD test was used.

Despite the fact that the HTC value in 1992 was different on both experimental trials, an attempt was made to compare the drought resilience components of the studied larch provenances between two trials. All provenances growing at BL were significantly higher (*p* < 0.05) resistant to the drought (Rt) than those at SI (Table 2). Most of the provenances originating from the Northern Poland, the Carpathians and the Sudety Mts. (nos. 1, 6, 14, 16, 18, 2, 22, 23 and 24) growing at SI were significantly (*p* < 0.05) higher recovered (Rc) after the drought than those at BL. The resilience index for all provenances growing at BL, except for no. 14 and 16, was significantly (*p* < 0.05) higher than in those at SI.

**Table 2:** Differences (– negative, + positive) between the values of the Rt, Rc, RS and RRs index after the 1992 drought for the larch provenances in the trials in SI and BL (SI values were subtracted from BL). Significant values of differences (*p* < 0.05) – brackets.

	Provenace number																			
Index	1	2	4	6	7	8	9	10	11	12	13	14	16	18	19	20	21	22	23	24
Rt	(-)	-	(-)	(-)	(-)	(-)	(-)	(-)	(-)	-	-	(-)	(-)	(-)	(-)	(-)	-	(-)	(-)	(-)
Rc	(+)	(-)	+	(+)	+	+	+	+	+	+	(-)	(+)	(+)	(+)	(+)	(+)	+	(+)	(+)	(+)
Rs	(-)	(-)	(-)	(-)	(-)	(-)	(-)	(-)	(-)	(-)	(-)	-	-	(-)	(-)	(-)	(-)	(+)	(-)	(-)
RRs	-	(-)	-	-	-	-	-	(-)	-	(-)	(-)	-	(+)	-	-	(+)	(-)	-	-	-

## Discussion

### Specificity of radial growth features of larch provenances at experimental trials

The results support the hypothesis that larches, regardless of their origin, show specific radial growth sensitivity to thermal and moisture conditions prevailing in a given trial. The different climatic conditions prevailing in the experimental trials located in the Polish lowlands (SI) and uplands (BL) had a very strong and specific influence on the radial growth responses of larch provenances growing there. The genotype, shaped over many years by, among other things, the specific climate features of their origin region (local adaptation) were dominated by the climatic conditions of the trials so strongly that they were not reflected in their radial growth reactions. Similar observations in the case of other species are also reported by other researchers. This was the case, for example, with Scots pine provenances from entire European, natural distribution range growing at 2 experimental trials in the Western and Northeastern Germany^26^, and with the Scots pine provenances from the entire Asian range growing at a trial in the Central Siberia in Russia^21^. Similar conclusions apply to Norway spruce provenances from the entire European range growing at 8 trials in various parts of Finland^28^. The study on Douglas fir provenances from native range in North America growing at 3 trials located in different climatic zones in the German Alps did not show a relationship between the origin region of trees and their growth decline in the extreme dry and hot year 2003 at the trials^24^.

Therefore, it was not possible to distinguish larch provenances in terms of the characteristics of their pattern of interannual radial growth variations, which would come from a specific, climatically homogeneous region, i.e. from lowlands, uplands or mountains. It turned out that the separate groups with specific radial growth pattern included provenances from various climatic regions of Poland. Differences in wood properties (elasticity, proportion of sapwood and heartwood, compressive and bending strength) and the growth dynamics parameters (maximum growth rate and growth acceleration rate) of larch at SI trial had also no genetic basis^66–70^. However, Wilczyński and Kulej^71^ distinguished two groups of North American giant fir provenances with the specific features of radial growth pattern. They were grown at trials in the Polish Carpathian Mountains. The first group consisted of giant firs from coastal areas and the second from mountainous areas of the North America. Thus, in this case, the influence of genome, which was formed in two native regions with strongly different climates, on their radial growth response after an introduction to the Polish mountains was clearly marked.

### Sensitivity of larch provenances to the thermal factor of experimental trials

A factor differentiating the radial growth reactions of larches between the experimental trials were a climatic conditions, which determined the proportion of trees with a specific growth reaction (decline and increase). About 1/3 more pointer years were in the upland trial (BL) than in the lowland trial (SI). This is puzzling, because climatic conditions in BL were more optimal for larch growth than in SI. More negative pointer years than positive were in the both trials. This suggests that weather conditions for larch growth were more frequent unfavourable than favourable.

Cold and dry February and cold March in the both trials adversely affected the wood increment of almost all larch provenances. Particularly strong relationships between the wood increment and the air temperature in the second half of winter season are visible in the case of larches at the trials located in the uplands (BL), where winters are colder and longer than at SI. Koprowski^44^ and Danek et al.^48^ also showed that local partial populations that were grown in the various parts of Poland were sensitive to cold and long winters. These conditions resulted in a shorter growing season which caused the increase in radial growth. However, the opposite reaction was observed in some provenances. For example, at SI, the increase in the growth of lowland larch provenances (nos. p1, p7 and p9) was negatively affected by high air temperature in February. At BL the larches of lowland provenance (no. p1) and came from the Sudetes (no. p22) decreased their growth when temperature in January was high. It is likely that these property may be genetically determined, as provenance no. p22 came from the Sudety Mts., where winter is relatively frosty and snowy. Another explanation is that when individual populations are moved to a different location, the response of larches to a given climatic element may change due to genotype-environment interaction^20^. Therefore, each provenance should be analyzed in detail in this respect.

Thus, a projected increase in temperature during the winter season^2,72^ may inhibit the radial growth of some larch provenances, but for others this effect will be positive and will result in an increase in their incremental vitality. Cambium activation in trees begins earlier, when winter ends sooner^73,74^. This contributes to wider wood rings deposited by larches^44,45,75,76^. Our results also confirm this. Warm March and April at SI and warm March at BL were conducive to the generation of wide wood rings by most larch provenances. However, some provenances reacted negatively to high temperature in April by reducing the radial growth.

The relationships between summer temperature and the growth of larch provenances were highly diverse at both trials. At SI, some western and mountain Polish provenances reacted positively to high temperature in June (no. p24), July (nos. p7, p20, p23, p24), August (nos. p23, p24) and September (nos. p7, p20, p23). At BL, also some upland’s and Sudeten provenances (nos. p19, p21, p24) increased wood growth when August was relatively warm.

### Sensitivity of larch origins to the pluvial factor

On the basis of the IT index, we found that the radial growth response of larch provenance to thermal-pluvial conditions in the autumn proceeding the year of ring formation was more diverse at the SI experimental trial than at BL. At the BL trial, a cold and abundant precipitation in September caused that the growth reaction of the vast majority of provenance was the increase in the tree-ring growth in the following year. European larch forms buds mainly in September^77^. Abundant precipitation, which is associated with high cloudiness and solar radiation deficit, causes that the vegetative buds are formed in trees. As a result, new assimilation apparatus (needles) and shoots are abundant in the following year, resulting in increased wood production^78^. There are therefore fewer flower buds and, consequently, fewer cones in the following year^79^. We also found that high precipitation in November at BL had a positive effect on the radial growth of all larch provenances in the following year. The accumulation of water reserves in the soil in autumn protects trees from winter frosts and has a beneficial effect on larch growth in the following year^45^. Also, high precipitation at both experimental trials in the end of winter (February) had a positive effect on the amount of wood formed by most of the larch provenances in the coming growing season. According to Rötzer et al.^80^, available soil water in the end of winter is necessary for the activation of tree cambium.

At the SI trial with relatively low precipitation totals, the vast majority of larch provenances deposited wide tree-rings when spring (March-May) was abundant in precipitation. However, at the trial located in the uplands (BL), most of the larch provenances decreased the radial growth when there was too much precipitation in spring. A lot of precipitation in spring also indicates high cloudiness, which lowers the air temperature, on which the activation of the vascular cambium depends.

We also found that larches of most provenances at the upland’s trial BL increase wood growth when summer precipitation is high. Although northern and Sudeten Polish provenances (e.g. nos. p2, p21) were slightly less sensitive to this climatic element. At the trial SI located in lowland with lower precipitation, e.g. the Sudeten provenance (no. p23) was slightly less demanding in relation to summer precipitation.

The above-described climate-radial growth relationships of different larch provenances indicate a large interpopulation diversity in terms of sensitivity to air temperature and precipitation. These relationships had no clear connection with the regions of larch seed source. This statement corresponds well with previous studies based on the correlation analysis of the size of wood growth and climatic parameters^42,43^. Some larch populations are not bothered by episodic precipitation shortages during the growing season. However, it is surprising that this applies to trees that come from regions with relatively high precipitation. Isohydric species (i.e. Norway spruce) reduce water consumption and growth already in the early phase of drought by closing the stomata. However, European larch, like beech and oak, is considered an anisohydrological tree species that does not exhibit restrictive stomatal regulation^35,37^. During droughts, larch still maintains high photosynthetic activity^45^. It should be emphasized that larch does not reduce the sap flow in the steam, including water, during the deficit of water vapour in the air and water in the soil during short summer droughts, and larch does not show a permanent reduction in growth during the summer^35^.

### Sensitivity of larch to drought

The reductions in radial growth of the studied larch provenances were associated with precipitation deficits occurring in different periods of the growing season in 1992. Climate diagrams and the index of climate dryness (HTC) showed that these periods were particularly dry compared to those in the three previous and three following years that were optimal. Therefore, in our case, the conditions in the three years preceeding the year with drought could not significantly disrupt the values of the resilience components. This gives such an indication for future research that it is necessary to carefully analyse weather conditions in previous years in order to fully assess the response of trees to drought. Droughts in Poland since 1951 have varied in severity, duration and location. They did not occur in both experimental trials we studied. However, the most severe drought with the greatest extent occurred in 1992. The long-lasting drought in 1992 took on the character of a natural disaster that covered the entire area of Poland, with the most severe occurrence in the northwestern and central parts of the country (i.e., the area in SI and BL). In some regions it even lasted for the entire growing season (April-September)^81,82^. Therefore, the comparison of differences between provenances in terms of drought resilience components in the 1992 example is highly authoritative.

It turns out that a given population on a given experimental trial showed a high variability between the indices and high variation in a given index between the trials. None of the provenances growing at the two experimental trials was fully drought tolerant (resistance index Rt < 1). Droughts often defoliate tree crowns^18,83^, and consequently reduce tree-ring growth^84^. Tree growth disorders caused by drought promote secondary damage in trees by insects or fungi^11^. However, extreme droughts can accelerate the mortality process, mainly in low-growing trees^85,86^.

The resistance index allows to indicate the most and least drought resistant larch provenances. At SI, the highest drought resistance index was found in the provenances nos. p8, p20, and the lowest in provenance no. p2. It is likely that these properties may be genetically determined, as provenances nos. p7, p8, p20 came from warm and relatively dry lowland’s regions of Poland, while origin no. p2 came from relatively cool and precipitation abundant northern part of Poland. At BL, the northern Polish provenance (no. p4) was the second lowest resistance to drought after Sudeten provenance (no. p21), and the highest drought resistance was provenance from the center of Polish uplands (no. p14).

At both trial, all provenances well recovered growth after drought (Rc > 1). At SI, provenances from the northern half of Poland (no. p2) recovered the growth best, and at BL it was origin no. p6. However, provenances from the southern half of the country were the weakest in the growth recovering. At SI it was provenance no. p20, and at BL no. p18.

Of all the provenances, only the 7 provenances at SI and BL had a high resilience of response to adverse climatic conditions. It showed a higher increase after drought than in the pre-drought period (Rs > 1). This may be due to the fact that this provenance came from the driest area of Poland with negative water balance for many years^2^. Even a slight improvement in water conditions in the years following the drought could have had a positive impact on the radial growth of this larch provenance. The least resilient growth reactions at SI and BL (Rs < 1 – lower growth after drought than in the pre-drought period) were found in the Carpathian provenance no. 16, originating from mountainous terrain which is quite abundant in precipitation. These results are hard to interpret at this stage of research.

After a drought, the provenances nos. p4, p6, p9, p13, p14, p16, p20, p21 at SI and provenances nos. p2, p9, p10, p12, p12, p19 at BL maintained the radial growth at a similar level as in the pre-drought period but reduced by the size of the growth level during drought (relative resilience index 1 < RRs > 1). The radial growth in the rest of the provenance was at a higher level after drought (RRs > 1). At SI, the lowest RRs represented provenance no. p16, and at BL no. p20, i.e. from the southern of Poland. Such a low radial growth rate may be related to the fact that anisohydric species transpire and grow despite drought stress until the water is depleted. They risk morphological changes, loss of tiny roots and leaves due to previous cavitation of water-conducting bundles as a result of drought stress. The latter means slower recovery from drought stress, because the growth can only be accelerated again after a tree overcame the xylem tissue cavitation and lost damaged organs^87^. However, in the case of extreme droughts, deeper root systems can use water from a larger volume of soil and thus delay the onset of water stress during drought^80^ which may have resulted in maintaining the radial growth for most larch provenances.

The analysis of the above-mentioned indices showed a high variability radial growth features in European larch trees of the same provenance between the experimental trials. However, within a given trial, no provenance was clearly marked by better or worse the all resilience components to all other provenances. Therefore, it was not possible to identify with absolute certainty the most suitable and future-oriented populations in terms of radial growth and resilience components for the region in which the trials were located.

## Conclusions

The research showed a variation in the annual growth rhythm of European larch trees at lowland and upland experimental trials. The grouping obtained in the study, concerning both the IT chronology and the series of climate-radial growth correlation coefficients, confirmed unambiguously the climatic causes of differences between provenances in terms of their sensitivity to the climatic factor which was characteristic for a given trial (region).

Rainwater reserves in the soil accumulated in the autumn of the previous year and the length of the growing season modelled by its beginning date had a significant impact on the amount of wood formed by larches, regardless of its origin. At both trials, the beginning of vegetation is determined by thermal conditions, mainly in February. In terms of the amount of wood deposited by larches, the thermal-pluvial conditions of the summer were also important. The colder and more abundant precipitation was in the region, the more homogeneous the radial growth reactions of the trees of the studied provenance to precipitation deficit and high temperature.

The variation of the radial growth responses to thermal and pluvial conditions, including droughts, was not related to the climatic regions from which larches originated. At two experimental trials, larches with the lowest and highest resistance, recovery and resilience came from different regions of Poland, and not, as assumed, from the wettest and driest regions, respectively. The differences in above-mentioned features between the provenances were usually statistically insignificant. This may indicate that the heritability of drought resilience components among the progeny of larches is low or that larch shows high plasticity in relation to the climatic conditions in which it currently grows.

The highest resistance to drought was observed in larch provenances growing at the trial BL located in the uplands, and not, as assumed, at the trial SI located in the lowlands. This may mean that larches, regardless of their origin, react badly to dry summers in the lowlands, i.e. where there is a frequent deficit of precipitation.

Larches growing in the lowlands presented lower relative resilience. This may indicate that larches, regardless of their origin, do not adapt well to dry periods in the lowlands. However, this adaptation is better in the uplands, because it is more common for a dry year to be followed by years with abundant precipitation.

The variability of the values of the above-mentioned indices in larches within the same provenance was high, what indicates a large individual genetic variability determining the sensitivity of trees to drought. The relative resilience index seems to be the most helpful in predicting the future incremental reactions of the studied larches to unfavorable growth conditions, and consequently predicting their viability and ability to survive in unfavorable conditions. It turned out that the variability of this indicator among individuals of a given provenance was the highest and its values were most often significantly different when comparing the pairs of studied larch provenances.

## Data Availability

The datasets generated during and/or analysed during the current study are available from the corresponding author on reasonable request.

## References

[1] Battisti, D. S. & Naylor, R. L. Historical warnings of future food insecurity with unprecedented seasonal heat. *Science* **323**, 240–244 (2009). 19131626 10.1126/science.1164363

[2] Wibig, J. et al. Dynamiczne scenariusze zmian klimatu dla Polski na Lata 2011–2030 in Warunki Klimatyczne i Oceanograficzne w Polsce i na Bałtyku Południowym – Spodziewane Zmiany i Wytyczne do Opracowania Strategii Adaptacyjnych w Gospodarce Krajowej (eds (eds Wibig, J. & Jakusik, E.) 93–123 (IMiGW-PIB, (2012).

[3] IPCC. Climate Change : The Physical Science Basis. Contribution of Working Group I to the Sixth Assessment Report of the Intergovernmental Panel on Climate Change (Cambridge University Press, 2021). (2021).

[4] Füssel, H-M. et al. *Climate Change, Impacts and Vulnerability in Europe* (EEA, 2012).

[5] Albert, M. et al. Assessing risks and uncertainties in forest dynamics under different management scenarios and climate change. *Ecosyst.* **2**, 14. 10.1186/s40663-015-0036-5 (2015).

[6] Ciais, P. et al. Europe-wide reduction in primary productivity caused by the heat and drought in 2003. *Nature* **437**, 529–533 (2003). 10.1038/nature03972 16177786

[7] Hartmann, H. Will a 385 million year-struggle for light become a struggle for water and for carbon? – how trees may cope with more frequent climate change-type drought events. *Glob Change Biol.* **17**, 642–655 (2010).

[8] Pretzsch, H. & Dieler, J. The dependency of the size-growth relationship of Norway spruce (*Picea abies* [L.] Karst.) And European beech (*Fagus sylvatica* [L.]) in forest stands on long-term site conditions, drought events, and ozone stress. *Trees* **25**, 355–369 (2011).

[9] McDowell, N. et al. Mechanisms of plant survival and mortality during drought: Why do some plants survive while others succumb to drought? *New. Phytol*. **178**, 719–739 (2008). 18422905 10.1111/j.1469-8137.2008.02436.x

[10] Allen, C. D. et al. A global overview of drought and heat-induced tree mortality reveals emerging climate change risks for forests. *Ecol. Manag*. **259**, 660–684 (2009).

[11] Griess, V. C. & Knoke, T. H. Growth performance, windthrow, and insects: Meta-analyses of parameters influencing performance of mixed-species stands in boreal and northern temperate biomes. *Can. J. Res.* **41**, 1141–1158 (2011).

[12] Merlin, M. et al. Effects of stand composition and tree size on resistance and resilience to drought in sessile oak and scots pine. *Ecol. Manag*. **339**, 22–33 (2015).

[13] Sacewicz, W. A. & Bijak, S. Wpływ trzebieży na przyrost radialny dębu w Nadleśnictwie Międzyrzec. *Sylwan* **163** (8), 645–654 (2019).

[14] Bolte, A. et al. Adaptive Forest Management, a prerequisite for sustainable forestry in the Face of Climate Change in Sustainable Forest Management in a Changing World, a European Perspective. (ed Spathelf, P.) 114–140 (Springer, (2010).

[15] Vitali, V., Büntgen, U. & Bauhus, J. Silver fir and Douglas fir are more tolerant to extreme droughts than Norway spruce in south-western Germany. *Glob Change Biol.* **23**, 5108–5119 (2017). 10.1111/gcb.13774 28556403

[16] Rahman, M., Islam, M. & Bräuning, A. Species-specific growth resilience to drought in a mixed semi-deciduous tropical moist forest in South Asia. *Ecol. Manag*. **433**, 487–496 (2019).

[17] Zang, C., Pretzsch, H. & Rothe, A. Size-dependent responses to summer drought in scots pine, Norway spruce and common oak. *Trees* **26**, 557–569 (2012).

[18] Lloret, F., Keeling, E. G. & Sala, A. Components of tree resilience: Effects of successive low–growth episodes in old Ponderosa pine forests. *Oikos* **120**, 1909–1920 (2011).

[19] Bruchwald, A. et al. Kształtowanie się przyrostu grubości jodeł z Gór Świętokrzyskich. *Sylwan* **160** (11), 893–904 (2016).

[20] Burczyk, J. & Giertych, M. Response of Norway spruce (*Picea abies* (L.) Karst.) Annual increments to drought for various provenances and locations. *Silvae Genet.* **40** (3–4), 146–152 (1991).

[21] Savva, Y. V. et al. Sensitivity of diameter growth to annual weather conditions in scots pine provenances at a central siberian location. *Silvea Genet.* **51** (2–3), 49–55 (2002).

[22] Savva, Y. et al. Effect of interannual climate variations on radial growth of jack pine provenances in Petawawa, Ontario. *Can. J. Res.* **38**, 619–630 (2008).

[23] McLane, S. C., Daniels, L. D. & Aitken, S. N. Climate impacts on lodgepole pine (*Pinus contorta*) radial growth in a provenance experiment. *Ecol. Manag*. **262**, 115–123 (2011).

[24] Jansen, K. et al. Tree ring isotopic composition, radial increment and height growth reveal provenance-specific reactions of Douglas-fir towards environmental parameters. *Trees* **27**, 37–52 (2012).

[25] Eilmann, B. et al. Origin matters! Difference in drought tolerance and productivity of coastal Douglas-fir (*Pseudotsuga menziesii* (Mirb.)) Provenances. *Ecol. Manag*. **302**, 133–143 (2013).

[26] Taeger, S. et al. Impact of climate and drought events on the growth of scots pine (*Pinus sylvestris* L.) provenances. *Ecol. Manag*. **307**, 30–42 (2013).

[27] Klisz, M. et al. Radial growth variation between four provenances of Norway spruce in the conditions of central Poland. *Res. Pap*. **76** (1), 59–65 (2015).

[28] Suvanto, S. et al. Geographical patterns in the radial growth response of Norway spruce provenances to climatic variation. *Agric. Meteorol.* **222**, 10–20 (2016).

[29] Nagamitsu, T., Matsuzaki, T. & Nagasaka, K. Provenance variations in stem productivity of 30-year-old Japanese larch trees planted in northern and central Japan are associated with climatic conditions in the provenances. *J. for. Res-Jpn*. **23** (5), 270–278 (2018).

[30] Schweingruber, F. H. et al. Identification, presentation and interpretation of event years and pointer years in dendrochronology. *Dendrochronologia* **8**, 9–38 (1990).

[31] Neuwirth, B., Schweingruber, F. H. & Winige, M. Spatial patterns of central European pointer years from 1901 to 1971. *Dendrochronologia* **24**, 79–89 (2007).

[32] Vitas, A. & Žeimavičius, K. Regional Tree-Ring Chronology of European Larch (*Larix decidua* Mill.) In Lithuania. *Balt for.* **16** (2), 187–193 (2010).

[33] Vitas, A. A. Dendroclimatological Analysis of European Larch (*Larix decidua* Mill.) From Lithuania. *Balt for.* **20** (1), 65–72 (2015).

[34] Wilczyński, S., Szymański, N. & Olejnik, M. Adaptacja wybranych pochodzeń modrzewia europejskiego do klimatu nizin centralnej Polski. *Sylwan* **160** (8), 656–665 (2016).

[35] Anfodillo, T. et al. Tree water relations and climatic variations at the alpine timberline: seasonal changes of sap flux and xylem water potential in *Larix decidua* Miller, *Picea abies* (L.) Karst, and *Pinus cembra* L. *Ann. Des. Sci. For.* **55**, 159–172 (1998).

[36] Schuster, R. & Oberhuber, W. Drought sensitivity of three cooccurring conifers within a dry inner Alpine environment. *Trees* **27**, 61–69 (2013). 23976821 10.1007/s00468-012-0768-6 PMC3750198

[37] Leo, M. R. et al. Evaluating the effect of plant water availability on inner alpine coniferous trees based on sap flow measurements. *Eur. J. Res.* **133**, 691–698 (2014).

[38] Obojes, N. et al. Water stress limits transpiration and growth of European larch up to the lower subalpine belt in an inner-alpine dry valley. *New. Phytol*. **220**, 460–475 (2018). 30028013 10.1111/nph.15348 PMC6586014

[39] Sasani, N. et al. Physiological and anatomical responses to drought stress differ between two larch species and their hybrid. *Trees* **35**, 1467–1484 (2021). 34720435 10.1007/s00468-021-02129-4 PMC8550302

[40] Szeligowski, H. Proweniencyjne różnice w odporności modrzewia europejskiego (*Larix decidua* Mill.) Na suszę. *Sylwan* **14**, 65–78 (2001).

[41] Wilczyński, S. B. & Kulej, M. The influence of climate on the radial increment of larches of different provenances on the basis of the experiment in the Carpathian Mountains in Southern Poland. *Eur. J. for. Res.* **132** (5-6), 919–929 (2013).

[42] Szymański, N. & Wilczyński, S. Radial growth response of European larch provenances to interannual climate variation in Poland. *Forests* 12, 334; (2021). 10.3390/f12030334

[43] Szymański, N. & Wilczyński, S. Wrażliwość drzew modrzewia europejskiego na warunki siedliskowe powierzchni doświadczalnych w Siemianicach, Bliżynie i Krynicy. *Sylwan* **165** (4), 324–335 (2021).

[44] Koprowski, M. Long-term increase of March temperature has no negative impact on tree-rings of European larch (*Larix decidua*) in lowland Poland. *Trees* **26**, 1895–1903 (2012).

[45] Lévesque, M. et al. Drought response of five conifer species under contrasting water availability suggests high vulnerability of Norway spruce and European larch. *Glob Change Biol.* **19** (10), 3184–3199 (2013). 10.1111/gcb.12268 23712589

[46] Danek, M., Chuchro, M. & Walanus, A. Variability in larch (*Larix decidua* Mill.) Tree-ring growth response to climate in the Polish Carpathian Mountains. *Forests* **8** (10), 354. 10.3390/f8100354 (2017).

[47] Danek, M., Chuchro, M. & Walanus, A. Tree-ring growth of larch (*Larix decidua* Mill.) In the Polish sudetes – the influence of altitude and site-related factors on the climate–growth relationship. *Forests* **9** (11), 663. 10.3390/f9110663 (2018).

[48] Danek, M., Chuchro, M. & Danek, T. Extreme growth reaction of larch (*Larix decidua* Mill.) From the Polish sudetes and carpathians: spatial distribution and climate impact. *Trees* **35**, 211–229 (2021).

[49] Danek, M. & Danek, T. Recent changes in the climate–growth response of European larch (*Larix decidua* Mill.) In the Polish sudetes. *Trees* **36**, 803–817 (2022).

[50] Szymański, N. & Wilczyński, S. Regiony dendroklimatyczne modrzewia europejskiego (*Larix decidua* Mill.) W Polsce. *Pr Geogr.* **169**, 69–85 (2022).

[51] Pretzsch, H., Schütze, G. & Uhl, E. Resistance of European tree species to drought stress in mixed versus pure forests: Evidence of stress release by inter – specific facilitation. *Plant. Biol.* **15** (3), 483–495 (2012). 23062025 10.1111/j.1438-8677.2012.00670.x

[52] Vilà-Cabrera, A. & Jump, A. S. Greater growth stability of trees in marginal habitats suggests a patchy pattern of population loss and retention in response to increased drought at the rear edge. *Ecol. Lett.* **22**, 1439–1448 (2019). 31250529 10.1111/ele.13329

[53] Zeng, X. et al. Qinghai spruce (*Picea Crassifolia*) and Chinese pine (*Pinus tabuliformis*) show high vulnerability and similar resilience to early-growing-season drought in the Helan Mountains, China. *Ecol. Indic.* **110**, 105871. 10.1016/j.ecolind.2019.105871 (2020).

[54] Bose, A. K. et al. Climate sensitivity and drought seasonality determine post-drought growth recovery of *Quercus petraea* and *Quercus robur* in Europe. *Sci. Total Environ.* **784**, 147222. 10.1016/j.scitotenv.2021.147222 (2021). 34088042 10.1016/j.scitotenv.2021.147222

[55] Jiao, L. et al. Comparison of the response stability of siberian larch to climate change in the Altai and Tianshan. *Ecol. Indic.* **128**, 107823. 10.1016/j.ecolind.2021.107823 (2021).

[56] Marcotti, E. et al. Growth resilience of *Austrocedrus chilensis* to drought along a precipitation gradient in Patagonia, Argentina. *Ecol. Manag*. **496**, 119–388 (2021).

[57] Chen, M. et al. Climate-growth pattern of *Pinus tabulaeformis* plantations and their resilience to drought events in the Loess Plateau. *Ecol. Manag*. **499**, 119642. 10.1016/j.foreco.2021.119642 (2021).

[58] Versace, S. et al. New evidence for population-specific responses to drought events from tree ring chronologies of *Pinus nigra* ssp. *laricio* across the entire distribution range. *Agric. Meteorol.* **499**, 119642. 10.1016/j.foreco.2021.119642 (2022).

[59] EUFORGEN. Distribution map of European Larch (*Larix decidua*) (2009). http://www.euforgen.org/species/larix-decidua

[60] Cybis, E. & Data, A. B. Technical writing, software development, photography and dendrochronology software (2024). https://www.cybis.se

[61] Holmes, R. L. Computer – assisted quality control in tree – ring dating and measurement. *Tree – Ring Bull.* 43, 69 – 78 (1983).

[62] Jetschke, G., van der Maaten, E. & van der Maaten-Theunissen, M. Towards the extremes: A critical analysis of pointer year detection methods. *Dendrochronologia* **53**, 55–62 (2019).

[63] Danielewicz, J. et al. Protection of Oats against Puccinia and Drechslera Fungi in various Meteorological conditions. *Appl. Sci.* **14**, 7121. 10.3390/app14167121 (2024).

[64] Hill, T., Lewicki, P. & Statistics Methods and Applications (StatSoft, (2006).

[65] Public data of Institute of Meteorology and Water Management National Research Institute. Poland https://danepubliczne.imgw.pl/ (2024).

[66] Szaban, J., Kowalkowski, W. & Jakubowski, M. Biegefestigkeit Von Holz Der Europaischen Lärche (*Larix decidua* Mill.) Auf Der Versuchsfläche Der Forstuntersuchungsanstalt LZD in Siemianice. *Ann. Wars Univ. Life Sci. Wood Technol.* **84**, 217–221 (2013).

[67] Szaban, J., Kowalkowski, W. & Jakubowski, M. Druckfestigkeit Von Längstfasern Der europäischen Lärche (*Larix decidua* Mill.) Verschiedener Provenienzen Auf Der Versuchsfläche Der LZD Siemianice. *Ann. Wars Univ. Life Sci. Wood Technol.* **84**, 213–216 (2013).

[68] Szaban, J., Jakubowski, M. & Kowalkowski, W. Moduł sprężystości przy zginaniu statycznym wybranych proweniencji modrzewia europejskiego. *Stud. Mat. CEPL w Rogowie*. **16** (39/2B), 161–170 (2014).

[69] Jakubowski, M. et al. Proportion of sapwood and heartwood in stems of European larch (*Larix decidua* Mill.) Origin from provenances experimental trial. *Ann. Wars Univ. Life Sci. Wood Technol.* **88**, 87–91 (2014).

[70] Jagiełło, R., Łukowski, A. & Kowalkowski, W. The Polish provenances of European larch overperform the expected growth dynamics indicated by the sigmoid model. *Forests* **13**, 1852. 10.3390/f13111852 (2022).

[71] Kulej, M. & Wilczyński, S. The growth of seven *Abies grandis* provenances in the climatic conditions of the Polish Carpathian Mountains. *Dendrobiology* **81**, 1–13 (2019).

[72] Falarz, M. *Climate Change in Poland: Past, Present, Future* (Springer Nature Switzerland AG, 2021).

[73] Rossi, S., Morin, H. & Deslauriers, A. Causes and correlations in cambium phenology: Towards an integrated framework of xylogenesis. *J. Exp. Bot.* **63**, 2117–2126 (2012). 22174441 10.1093/jxb/err423 PMC3295399

[74] Begum, S. et al. Regulation of cambial activity in relation to environmental conditions, understanding the role of temperature in wood formation of trees. *Physiol. Plant.* **147**, 46–54 (2013). 22680337 10.1111/j.1399-3054.2012.01663.x

[75] Oleksyn, J., Fritts, H. C. & Hughes, M. K. Tree-ring analysis of different *Pinus sylvestris* provenances, *Quercus robur*, *Larix deci*dua and *L. decidua* x *L. kaempferi* affected by air pollution. *Arbor. Kórn.* 38, 89–109 (1993).

[76] Oberhuber, W. et al. Radial stem growth in response to microclimate and soil moisture in a drought-prone mixed coniferous forest at an inner Alpine site. *Eur. J. for. Res.* **133**, 467–479 (2014). 24883053 10.1007/s10342-013-0777-z PMC4035765

[77] Owens, J. N. & Molder, M. Bud development in Larix occidentalis. II. Cone differentiation and early development. *Can. J. Bot.* **57**, 1557–1572 (1979).

[78] Chałupka, W., Giertych, M. & Królikowski, Z. The effect of cone crops on growth in scots pine on tree diameter increment. *Arbor Kórn*. **21**, 361–366 (1976).

[79] Fober, H. Relation between climatic factors and scots pine (*Pinus sylvestris*) cone crops in Poland. *Arbor Kórn*. **21**, 367–374 (1977).

[80] Rötzer, T., Grote, R. & Pretzsch, H. The timing of bud burst and its effect on tree growth. *Int. J. Biometeorol.* **48**, 109–118 (2004). 14564495 10.1007/s00484-003-0191-1

[81] Łabędzki, L. Problematyka susz w Polsce. *Woda Środ Obsz Wiej*. **4** (1), 47–66 (2004).

[82] Wibig, J. Warunki wilgotnościowe w Polsce w świetle wskaźnika standaryzowanego klimatycznego bilansu wodnego. *Woda Środ Obsz Wiej*. **2** (38), 329–340 (2012).

[83] Bréda, N. et al. Temperate forest trees and stands under severe drought: A review of ecophysiological responses, adaptation processes and long-term consequences. *Ann. Sci.* **63**, 625–644 (2006).

[84] Millar, C. I., Westfall, R. D. & Delany, D. L. Response of high-elevation limber pine (*Pinus flexilis*) to multiyear droughts and 20th-century warming, Sierra Nevada, California, USA. *Can. J. Res.* **37**, 2508–2520 (2007).

[85] Pedersen, B. S. The role of stress in the mortality of midwestern oaks as indicated by growth prior to death. *Ecology* **79**, 79–93 (1998).

[86] Galiano, L., Martínez-Vilalta, J. & Lloret, F. Drought-induced multifactor decline of scots pine in the pyrenees and potential vegetation change by the expansion of co-occurring oak species. *Ecosystems* **13**, 978–991 (2010).

[87] Jones, H. Stomatal control of photosynthesis and transpiration. *J. Exp. Bot.* **49**, 387–398 (1998).

