# Author Correction: The tree-ring width and interval trend values as indicators of sensitivity to temperature and precipitation in different provenances of European larch

**DOI:** 10.1038/s41598-025-92468-w

**Published:** 2025-03-06

**Authors:** Norbert Szymański, Sławomir Wilczyński, Jan Kowalczyk, Wojciech Kowalkowski

**Affiliations:** 1 Department of Forest Ecology and Silviculture, University of Agriculture in Krakow, al. 29 Listopada 46, 31-425 Krakow, Poland; 2 Department of Forest Ecosystem Protection, University of Agriculture in Krakow, al. 29 Listopada 46, 31-425 Krakow, Poland; 3 Department of Silviculture and Genetics of Forest Trees, Forest Research Institute, Sękocin Stary, Ul. Braci Leśnej 3, 05-090 Raszyn, Poland; 4 Department of Silviculture, Poznań University of Life Sciences, Ul. Wojska Polskiego 71A, 60-625 Poznań, Poland

Correction to: *Scientific Reports* 10.1038/s41598-025-85652-5, published online 11 January 2025

The Funding section in the original version of this Article was incomplete.

“This research was financed by the National Science Centre, Poland (grant number: 2015/19/N/NZ9/00625) and the subvention of the Ministry of Science and Higher Education for the University of Agriculture in Krakow for 2025.”

now reads:

“This research was financed by the National Science Centre, Poland (grant number: 2015/19/N/NZ9/00625) and the subvention of the Ministry of Science and Higher Education for the University of Agriculture in Krakow for 2025. The publication was co-financed by the Polish Minister of Science and Higher Education as part of the Strategy of the Poznan University of Life Sciences for 2024-2026 in the field of improving scientific research and development work in priority research areas.”

The original Article has been corrected.

